# Supplementary material for: Rac1/Wave2/Arp3 Pathway Mediates Rat Blood-Brain Barrier Dysfunction under Simulated Microgravity Based on Proteomics Strategy
Source: Int J Mol Sci. 2021 May 13;22(10):5165. doi: 10.3390/ijms22105165 (PMC8153163; doi:10.3390/ijms22105165)
Supplement: Supplementary file 1 [file ijms-22-05165-s001.zip › Supplemental FIGURE.pdf]

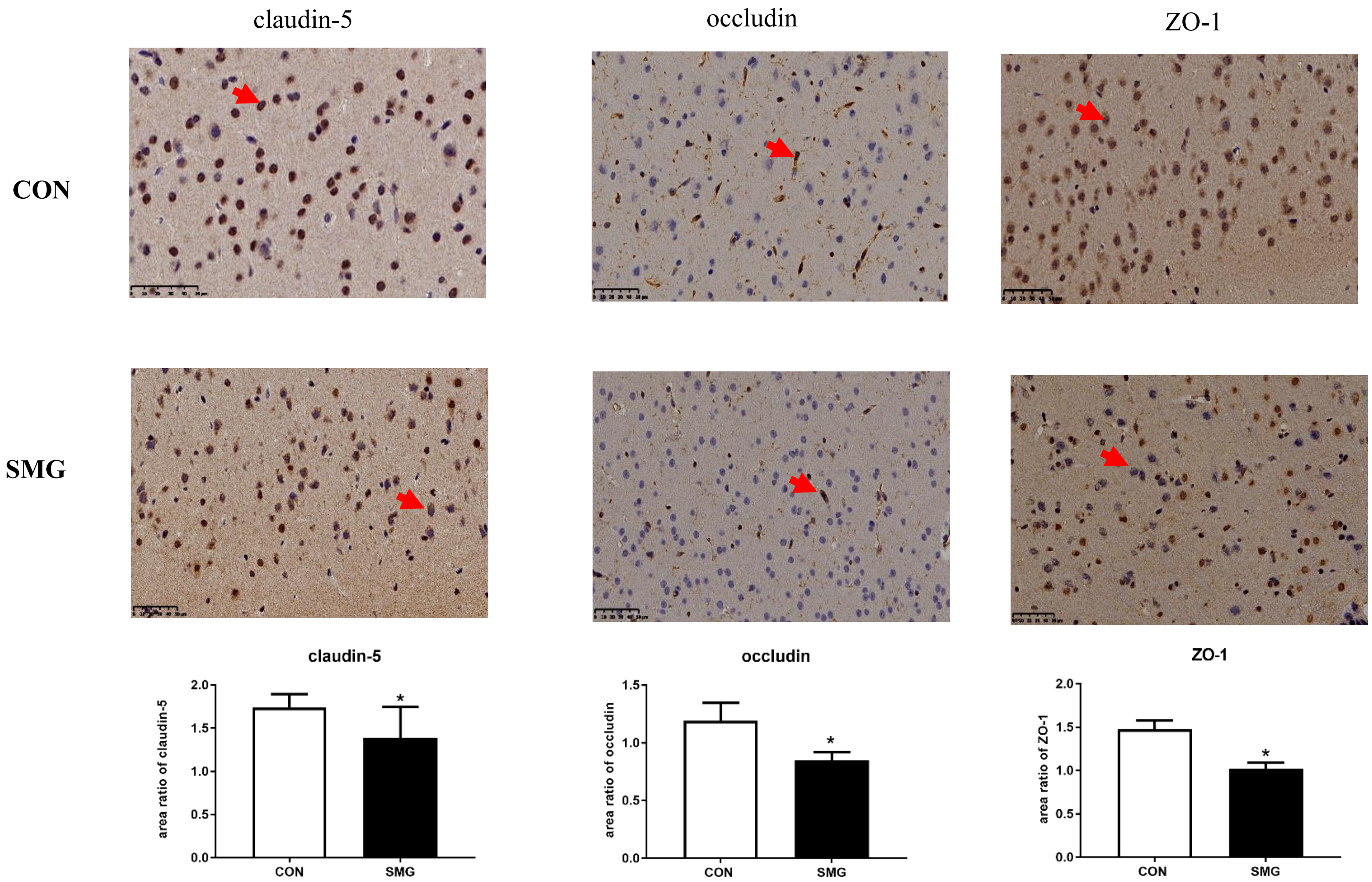

Supplementary Figure S1. Immunohistochemistry was performed to detect the expression and distribution of claudin-5, occludin and ZO-1 (arrows indicate the expression of TJs proteins) in brain of rat (scale bar, 50 μm). Claudin-5, occludin and ZO-1 expression significantly decreased under SMG conditions. SMG did not alter the distribution of TJs protein in the rat brain.

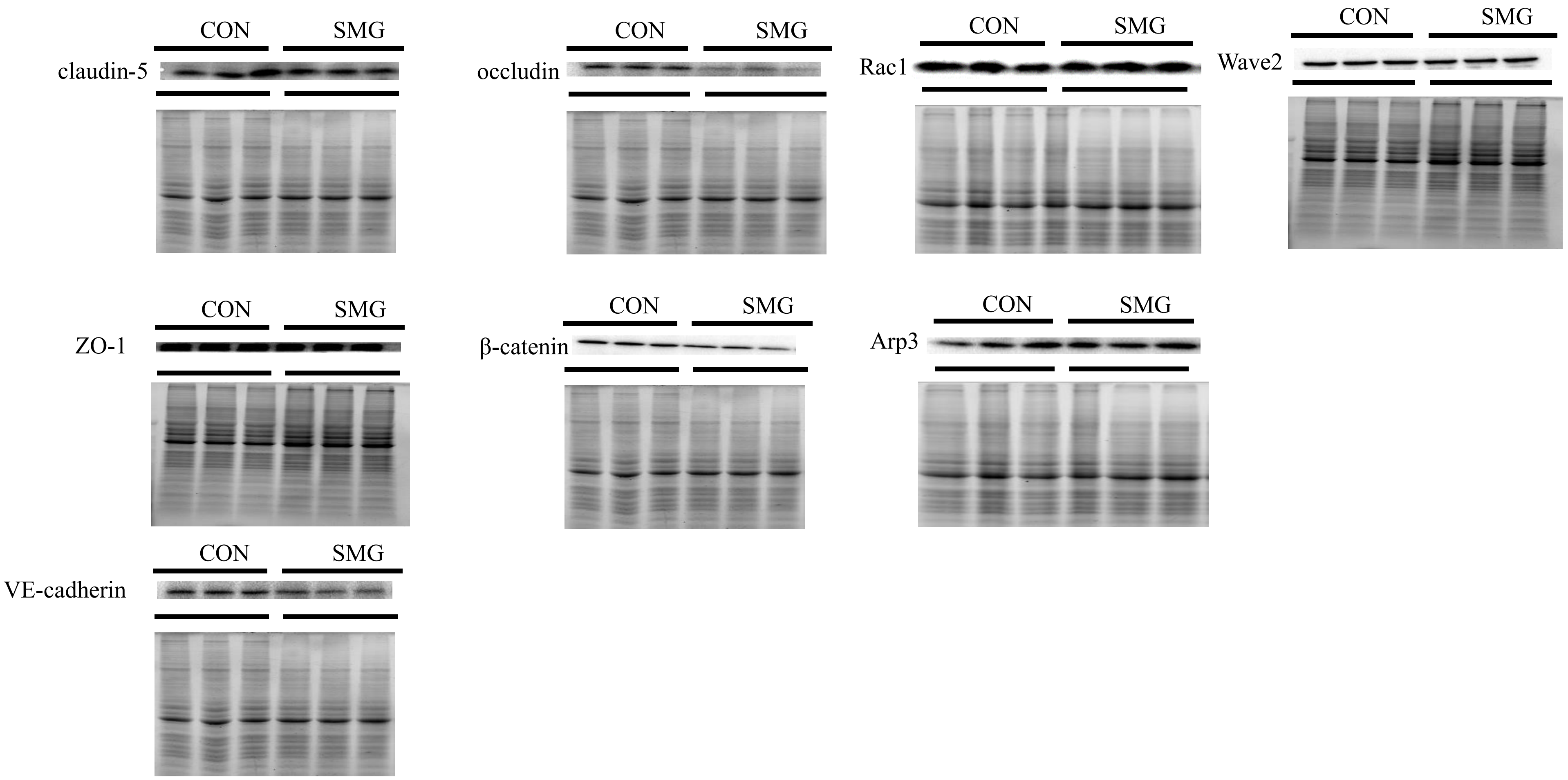

Supplementary Figure S2. Effects of SMG on protein expression of claudin-5, occluding, ZO-1, β-catenin, VE-cadherin, Rac1, Wave2 and Arp3 in HBMECs as determined by Western blot.

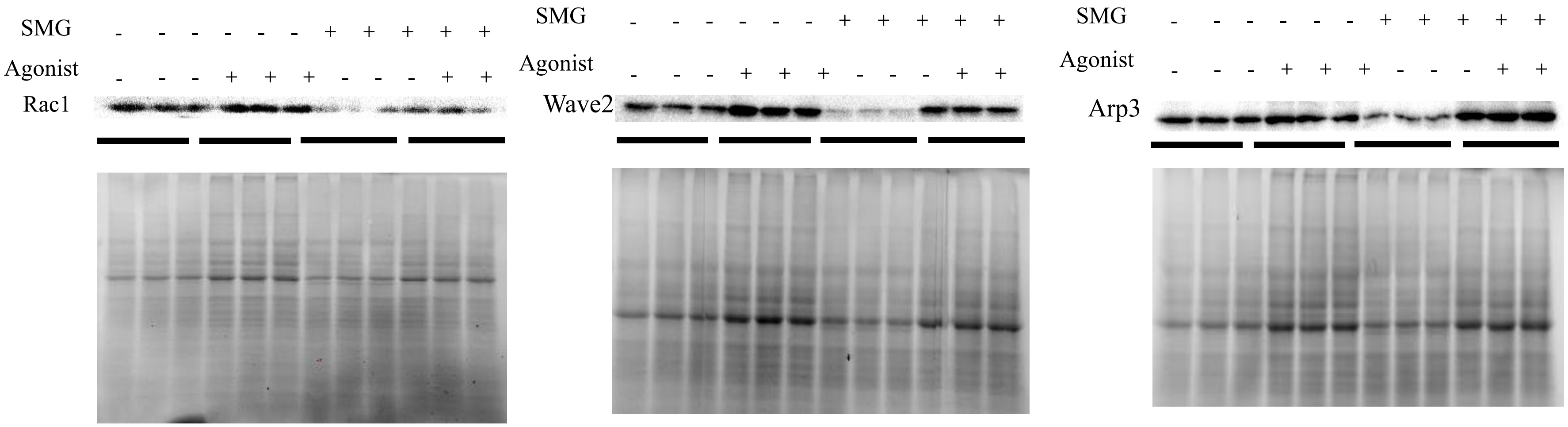

Supplementary Figure S3. HBMECs were pretreated with Rac1 agonist for 30 mins and then SMG for 24h , and the expression of Rac1, Wave2 and Arp3 were detected by Western blot.
