# Supplementary material for: Rac1/Wave2/Arp3 Pathway Mediates Rat Blood-Brain Barrier Dysfunction under Simulated Microgravity Based on Proteomics Strategy
Source: Int J Mol Sci. 2021 May 13;22(10):5165. doi: 10.3390/ijms22105165 (PMC8153163; doi:10.3390/ijms22105165)
Supplement: Supplementary file 1 [file ijms-22-05165-s001.zip › Supplemental Table.pdf]

Supplementary Table S1. The list of all differently expressed proteins

UniProt IDs	Protein names	Fold change		*P*-value
P14480	Fibrinogen beta chain	959.20	↑	0.0000
P01244	Somatotropin	573.01	↑	0.0041
P02680	Fibrinogen gamma chain	286.76	↑	0.0003
Q6AXR4	Beta-hexosaminidase subunit beta	13.37	↑	0.0220
Q7TP36	Protein Shroom2	12.37	↑	0.0016
P09215	Protein kinase C delta type	10.13	↑	0.0313
P02564	Myosin-7	10.05	↑	0.0165
Q9QYJ4	A TP-binding cassette sub-family B member 9	9.91	↑	0.0008
Q9Z1Z3	Epsin-2	9.24	↑	0.0004
Q9Z327	Synaptopodin	7.98	↑	0.0004
Q5XIG0	ADP-ribose pyrophosphatase, mitochondrial	7.46	↑	0.0232
P62961	Nuclease-sensitive element-binding protein 1	7.22	↑	0.0055
B0BNA7	Eukaryotic translation initiation factor 3 subunit I	6.04	↑	0.0148
Q4QQT4	Serine/threonine-protein phosphatase 2A 65 kDa regulatory subunit A beta isoform	5.70	↑	0.0001
Q5BJL5	Protein strawberry notch homolog 1	5.43	↑	0.0038
Q66H80	Coatomer subunit delta	5.23	↑	0.02134
P20673	Argininosuccinate lyase	4.68	↑	0.0013
Q64119	Myosin light polypeptide 6	4.61	↑	0.0266
O88377	Phosphatidylinositol 5-phosphate 4-kinase type-2 beta	4.44	↑	0.0170
P17105	Inositol-trisphosphate 3-kinase A	4.09	↑	0.0064
O88831	Calcium/calmodulin-dependent protein kinase kinase 2	4.07	↑	0.0115
O35867	Enolase-phosphatase E1	3.89	↑	0.0184
P12001	60S ribosomal protein L18	3.85	↑	0.0126
P18266	Glycogen synthase kinase-3 beta	3.76	↑	0.0083

Continued Table

UniProt IDs	Protein names	Fold change		*P*-value
Q9WVA1	Mitochondrial import inner membrane translocase subunit Tim8 A	3.63	↑	0.0013
Q9JI12	V esicular glutamate transporter 2	3.61	↑	0.0380
P62997	Transformer-2 protein homolog beta	3.47	↑	0.0182
O35964	Endophilin-A2	3.45	↑	0.0072
Q9ESH6	Glutaredoxin-1	3.43	↑	0.0024
Q6AXU6	Jupiter microtubule associated homolog 1	3.38	↑	0.0250
P49088	Asparagine synthetase [glutamine-hydrolyzing]	3.36	↑	0.0125
D3ZEF4	Cullin-7	3.35	↑	0.0057
Q64542	Plasma membrane calcium-transporting A TPase 4	3.34	↑	0.0002
Q62847	Gamma-adducin	3.29	↑	0.0179
Q4V898	RNA-binding motif protein, X chromosome	3.26	↑	0.0271
O54921	Exocyst complex component 2	3.26	↑	0.0013
P61459	Pterin-4-alpha-carbinolamine dehydratase	3.22	↑	0.0003
P63281	SUMO-conjugating enzyme UBC9	3.16	↑	0.0014
Q63151	Long-chain-fatty-acid--CoA ligase 3	3.01	↑	0.0079
Q8K1Q0	Glycylpeptide N-tetradecanoyltransferase 1	2.70	↑	0.0027
O88506	STE20/SPS1-related proline-alanine-rich protein kinase	2.66	↑	0.0126
Q5U2U2	Crk-like protein	2.66	↑	0.0489
P13086	Succinate--CoA ligase [ADP/GDP-forming] subunit alpha, mitochondrial	2.58	↑	0.0014
P97874	Cyclin-G-associated kinase	2.50	↑	0.0415
P11240	Cytochrome c oxidase subunit 5A, mitochondrial	2.47	↑	0.0138
Q62784	Type I inositol 3,4-bisphosphate 4-phosphatase	2.40	↑	0.0024
Q63615	Vacuolar protein sorting-associated protein 33A	2.37	↑	0.0010
O35458	V esicular inhibitory amino acid transporter	2.25	↑	0.0206

Continued Table

UniProt IDs	Protein names	Fold change		*P*-value
P62864	40S ribosomal protein S30	2.24	↑	0.0019
Q66HL2	Src substrate cortactin	2.21	↑	0.0000
P23514	Coatomer subunit beta	2.19	↑	0.0010
P27791	cAMP-dependent protein kinase catalytic subunit alpha	2.17	↑	0.0133
Q5XI22	Acetyl-CoA acetyltransferase, cytosolic	2.17	↑	0.0293
Q6P7B0	Tryptophan--tRNA ligase, cytoplasmic	2.17	↑	0.0057
Q6J4I0	Protein phosphatase 1 regulatory subunit 1B	2.17	↑	0.0440
Q9EPH2	MARCKS-related protein	2.16	↑	0.0235
P09216	Protein kinase C epsilon type	2.15	↑	0.0244
P04692	Tropomyosin alpha-1 chain	2.11	↑	0.0132
P10362	Secretogranin-2	2.11	↑	0.0050
P04785	Protein disulfide-isomerase	2.05	↑	0.0317
P23565	Alpha-internexin	1.99	↑	0.0067
G3V9R8	Heterogeneous nuclear ribonucleoprotein C	1.94	↑	0.0306
Q9JHL4	Drebrin-like protein	1.92	↑	0.0120
P70580	Membrane-associated progesterone receptor component 1	1.92	↑	0.0120
Q5FVM4	Non-POU domain-containing octamer-binding protein	1.92	↑	0.0325
Q63413	Spliceosome RNA helicase Ddx39b	1.91	↑	0.0325
Q6AXS5	Plasminogen activator inhibitor 1 RNA-binding protein	1.90	↑	0.0054
Q66H62	Ubiquitin carboxyl-terminal hydrolase CYLD	1.88	↑	0.0211
Q01205	Dihydrolipoyllysine-residue succinyltransferase component of 2-oxoglutarate dehydrogenase complex	1.83	↑	0.0032
P25809	Creatine kinase U-type, mitochondrial	1.82	↑	0.0300
Q64611	Cysteine sulfinic acid decarboxylase	1.82	↑	0.0484
B0BNF1	Septin-8	1.79	↑	0.0445

Continued Table

UniProt IDs	Protein names	Fold change		*P*-value
Q641Y8	A TP-dependent RNA helicase DDX1	1.78	↑	0.0010
Q07009	Calpain-2 catalytic subunit	1.76	↑	0.0413
Q04940	Neurogranin	1.75	↑	0.0413
P02650	Apolipoprotein E	1.74	↑	0.0113
Q9QXU9	ProSAAS	1.73	↑	0.0117
P11030	Acyl-CoA-binding protein	1.71	↑	0.0156
Q8CGV7	Thiamine-triphosphatase	1.69	↑	0.0017
Q8CF97	Deubiquitinating protein VCIP135	1.69	↑	0.0296
P54690	Branched-chain-amino-acid aminotransferase, cytosolic	1.64	↑	0.0398
P13596	Neural cell adhesion molecule 1	1.64	↑	0.0010
P21531	60S ribosomal protein L3	1.63	↑	0.0128
Q62940	E3 ubiquitin-protein ligase NEDD4	1.63	↑	0.0215
P13697	NADP-dependent malic enzyme	1.57	↑	0.0319
Q8VD52	Pyridoxal phosphate phosphatase	1.57	↑	0.0027
P10111	Peptidyl-prolyl cis-trans isomerase A	1.57	↑	0.0406
Q5XIJ6	BRISC and BRCA1-A complex member 1	1.53	↑	0.0009
P42930	Heat shock protein beta-1	1.53	↑	0.0038
P27653	C-1-tetrahydrofolate synthase, cytoplasmic	1.50	↑	0.0252
P04636	Malate dehydrogenase, mitochondrial	0.66	↓	0.0147
Q9EPH8	Polyadenylate-binding protein 1	0.66	↓	0.0202
P20171	GTPase HRas	0.66	↓	0.0122
P63319	Protein kinase C gamma type	0.66	↓	0.0049
Q62950	Dihydropyrimidinase-related protein 1	0.66	↓	0.0326
P68182	cAMP-dependent protein kinase catalytic subunit beta	0.65	↓	0.0165

Continued Table

UniProt IDs	Protein names	Fold change		*P*-value
A0JPJ7	Obg-like A TPase 1	0.65	↓	0.0063
Q4V7C6	GMP synthase [glutamine-hydrolyzing]	0.65	↓	0.0042
O35264	Platelet-activating factor acetylhydrolase IB subunit beta	0.65	↓	0.0279
Q6P4Z9	COP9 signalosome complex subunit 8	0.64	↓	0.0413
P63322	Ras-related protein Ral-A	0.64	↓	0.0301
P97526	Neurofibromin	0.64	↓	0.0374
O08557	N(G),N(G)-dimethylarginine dimethylaminohydrolase 1	0.64	↓	0.0047
P83868	Prostaglandin E synthase 3	0.63	↓	0.0152
P07335	Creatine kinase B-type	0.63	↓	0.0182
Q6RUV5	Ras-related C3 botulinum toxin substrate 1	0.63	↓	0.0478
Q9JK11	Reticulon-4	0.63	↓	0.0282
P30349	Leukotriene A-4 hydrolase	0.63	↓	0.0343
P97685	Neurofascin	0.63	↓	0.0380
O70196	Prolyl endopeptidase	0.63	↓	0.0059
P41498	Low molecular weight phosphotyrosine protein phosphatase	0.63	↓	0.0003
A7VJC2	Heterogeneous nuclear ribonucleoproteins A2/B1	0.62	↓	0.0024
P56574	Isocitrate dehydrogenase [NADP], mitochondrial	0.62	↓	0.0217
Q6PST4	Atlastin-1	0.62	↓	0.0173
Q6AYE2	Endophilin-B1	0.61	↓	0.0387
Q63537	Synapsin-2	0.61	↓	0.0276
P80254	D-dopachrome decarboxylase	0.61	↓	0.0154
Q91ZN1	Coronin-1A	0.61	↓	0.0123
Q91Z79	Liprin-alpha-3	0.60	↓	0.0389
P46413	Glutathione synthetase	0.60	↓	0.0247

Continued Table

UniProt IDs	Protein names	Fold change		*P*-value
P69682	Adaptin ear-binding coat-associated protein 1	0.60	↓	0.0247
Q07310	Neurexin-3	0.60	↓	0.0070
Q9QZA2	Programmed cell death 6-interacting protein	0.59	↓	0.0104
P08413	Calcium/calmodulin-dependent protein kinase type II subunit beta	0.59	↓	0.0192
P11884	Aldehyde dehydrogenase, mitochondrial	0.59	↓	0.0093
Q63716	Peroxiredoxin-1	0.59	↓	0.0117
Q99NA5	Isocitrate dehydrogenase [NAD] subunit alpha, mitochondrial	0.59	↓	0.0001
Q5XIF6	Tubulin alpha-4A chain	0.59	↓	0.0153
P13221	Aspartate aminotransferase, cytoplasmic	0.59	↓	0.0019
P30713	Glutathione S-transferase theta-2	0.59	↓	0.0076
P62138	Serine/threonine-protein phosphatase PP1-alpha catalytic subunit	0.59	↓	0.0098
P25113	Phosphoglycerate mutase 1	0.59	↓	0.0253
F1LMZ8	26S proteasome non-A TPase regulatory subunit 11	0.59	↓	0.0110
Q4QRB4	Tubulin beta-3 chain	0.58	↓	0.0191
P28073	Proteasome subunit beta type-6	0.58	↓	0.0217
P49621	Diacylglycerol kinase beta	0.58	↓	0.0403
Q64548	Reticulon-1	0.58	↓	0.0234
O35263	Platelet-activating factor acetylhydrolase IB subunit gamma	0.58	↓	0.0004
P62193	26S proteasome regulatory subunit 4	0.57	↓	0.0096
P62243	40S ribosomal protein S8	0.57	↓	0.0196
Q08163	Adenylyl cyclase-associated protein 1	0.57	↓	0.0034
Q08877	Dynamin-3	0.57	↓	0.0068
P15205	Microtubule-associated protein 1B	0.57	↓	0.0287
Q5FVI6	V-type proton A TPase subunit C 1	0.56	↓	0.0058

Continued Table

UniProt IDs	Protein names	Fold change		*P*-value
Q7TP47	synaptotagmin binding, cytoplasmic RNA interacting protein(Syncrip)	0.52	↓	0.0469
B2RZ78	VPS29 retromer complex component(Vps29)	0.52	↓	0.0347
P29314	ribosomal protein S9(Rps9)	0.51	↓	0.0044
Q62936	discs large MAGUK scaffold protein 3(Dlg3)	0.51	↓	0.0443
P18418	calreticulin(Calr)	0.51	↓	0.0009
P09117	aldolase, fructose-bisphosphate C(Aldoc)	0.51	↓	0.0005
Q8VBU2	NDRG family member 2(Ndrg2)	0.51	↓	0.0013
P34926	microtubule-associated protein 1A(Map1a)	0.51	↓	0.0114
Q3B8Q0	microtubule-associated protein, RP/EB family, member 2(Mapre2)	0.51	↓	0.0431
P81155	voltage-dependent anion channel 2(Vdac2)	0.51	↓	0.0011
P81795	eukaryotic translation initiation factor 2 subunit gamma(Eif2s3)	0.51	↓	0.0025
P35465	p21 (RAC1) activated kinase 1(Pak1)	0.51	↓	0.0462
P61314	ribosomal protein L15(Rpl15)	0.50	↓	0.0020
P36972	adenine phosphoribosyl transferase(Aprt)	0.50	↓	0.0015
Q566R0	thioesterase superfamily member 4(Them4)	0.50	↓	0.0271
P10719	ATP synthase, H+ transporting, mitochondrial F1 complex, beta polypeptide(Atp5b)	0.50	↓	0.0013
B2RYG6	OTU deubiquitinase, ubiquitin aldehyde binding 1(Otub1)	0.50	↓	0.0001
P31044	phosphatidylethanolamine binding protein 1(Pebp1)	0.50	↓	0.0012
P18666	myosin light chain 12B(Myl12b)	0.50	↓	0.0013
Q63016	solute carrier family 7 member 5(Slc7a5)	0.50	↓	0.0381
Q9R080	G-protein signaling modulator 1(Gpsm1)	0.50	↓	0.0084
P17220	proteasome subunit alpha 2(Psma2)	0.50	↓	0.0289
P97924	kalirin, RhoGEF kinase(Kalrn)	0.50	↓	0.0006
B0BNA5	coactosin-like F-actin binding protein 1(Cotl1)	0.50	↓	0.0474

Continued Table

UniProt IDs	Protein names	Fold change		*P*-value
B2DD29	BR serine/threonine kinase 1(Brsk1)	0.50	↓	0.0209
P50408	ATPase H+ transporting V1 subunit F(Atp6v1f)	0.50	↓	0.0067
Q07205	eukaryotic translation initiation factor 5(Eif5)	0.56	↓	0.0137
P51647	aldehyde dehydrogenase 1 family, member A1(Aldh1a1)	0.55	↓	0.0017
P27682	secretogranin V(Scg5)	0.55	↓	0.0321
Q5XFX0	transgelin 2(Tagln2)	0.55	↓	0.0005
P09456	protein kinase cAMP-dependent type 1 regulatory subunit alpha(Prkar1a)	0.55	↓	0.0383
Q06647	ATP synthase, H+ transporting, mitochondrial F1 complex, O subunit(Atp5o)	0.55	↓	0.0382
P62268	ribosomal protein S23(Rps23)	0.55	↓	0.0120
P62083	ribosomal protein S7(Rps7)	0.55	↓	0.0113
P12007	isovaleryl-CoA dehydrogenase(Ivd)	0.55	↓	0.0080
P00406	COXII(COX2)	0.54	↓	0.0053
P13233	2',3'-cyclic nucleotide 3' phosphodiesterase(Cnp)	0.54	↓	0.0027
P68511	tyrosine 3-monooxygenase/tryptophan 5-monooxygenase activation protein, eta(Ywhah)	0.54	↓	0.0041
P84076	hippocalcin(Hpca)	0.54	↓	0.0031
P49911	acidic nuclear phosphoprotein 32 family member A(Anp32a)	0.54	↓	0.0069
Q9JLZ1	glutaredoxin 3(Glrx3)	0.53	↓	0.0150
B0BND0	ectonucleotide pyrophosphatase/phosphodiesterase 6(Enpp6)	0.53	↓	0.0007
Q60587	hydroxyacyl-CoA dehydrogenase (trifunctional protein), beta subunit(Hadhb)	0.53	↓	0.0042
P85834	Tu translation elongation factor, mitochondrial(Tufm)	0.53	↓	0.0023
P06214	aminolevulinate dehydratase(Alad)	0.53	↓	0.0211
P17077	ribosomal protein L9(Rpl9)	0.52	↓	0.0029
Q5XI32	capping actin protein of muscle Z-line beta subunit(Capzb)	0.52	↓	0.0474

Continued Table

UniProt IDs	Protein names	Fold change		*P*-value
Q08602	Geranylgeranyl transferase type-2 subunit alpha	0.56	↓	0.0033
P41562	Isocitrate dehydrogenase [NADP] cytoplasmic	0.56	↓	0.0401
P97697	Inositol monophosphatase 1	0.56	↓	0.0119
P85971	6-phosphogluconolactonase	0.55	↓	0.0248
P85969	Beta-soluble NSF attachment protein	0.55	↓	0.0177
O55171	Acyl-coenzyme A thioesterase 2, mitochondrial	0.55	↓	0.0294
P97710	Tyrosine-protein phosphatase non-receptor type substrate 1	0.55	↓	0.0008
Q99N27	Sorting nexin-1	0.55	↓	0.0221
P63036	DnaJ homolog subfamily A member 1	0.55	↓	0.0382
Q5EB77	Ras-related protein Rab-18	0.55	↓	0.0120
Q5RKJ1	E3 ubiquitin-protein transferase MAEA	0.55	↓	0.0113
Q5XHY5	Threonine--tRNA ligase, cytoplasmic	0.55	↓	0.0080
P04904	Glutathione S-transferase alpha-3	0.54	↓	0.0053
Q5XI73	Rho GDP-dissociation inhibitor 1	0.54	↓	0.0027
P24368	Peptidyl-prolyl cis-trans isomerase B	0.54	↓	0.0041
B3GNI6	Septin-11	0.54	↓	0.0031
Q5M7A7	CB1 cannabinoid receptor-interacting protein 1	0.54	↓	0.0069
P22062	Protein-L-isoaspartate	0.53	↓	0.0150
Q9EQX9	Ubiquitin-conjugating enzyme E2 N	0.53	↓	0.0007
Q9Z0W5	Protein kinase C and casein kinase substrate in neurons protein 1	0.53	↓	0.0042
Q9WU70	Syntaxin-binding protein 5	0.53	↓	0.0023
P61265	Syntaxin-1B	0.53	↓	0.0211
Q9JJK1	Neuronal membrane glycoprotein M6-b	0.52	↓	0.0029
Q63797	Proteasome activator complex subunit 1	0.52	↓	0.0474

Continued Table

UniProt IDs	Protein names	Fold change		*P*-value
B0BN18	Prefoldin subunit 2	0.49	↓	0.0023
P61983	14-3-3 protein gamma [Cleaved into: 14-3-3 protein gamma, N-terminally processed]	0.49	↓	0.0003
Q9QYL8	Acyl-protein thioesterase 2	0.49	↓	0.0408
Q5RJP0	Aldose reductase-related protein 1	0.49	↓	0.0299
Q6P9V9	Tubulin alpha-1B chain	0.49	↓	0.0035
O70277	Tripartite motif-containing protein 3	0.48	↓	0.0056
P62271	40S ribosomal protein S18	0.48	↓	0.0008
P27139	Carbonic anhydrase 2	0.48	↓	0.0001
Q6Q0N1	Cytosolic non-specific dipeptidase	0.48	↓	0.0101
Q63692	Hsp90 co-chaperone Cdc37	0.48	↓	0.0076
P47819	Glial fibrillary acidic protein	0.47	↓	0.0079
Q71TY3	40S ribosomal protein S27	0.47	↓	0.0239
Q6IRE4	Tumor susceptibility gene 101 protein	0.47	↓	0.0060
P48679	Prelamin-A/C [Cleaved into: Lamin-A/C]	0.47	↓	0.0197
P07340	Sodium/potassium-transporting A TPase subunit beta-1	0.47	↓	0.0436
P35745	Acylphosphatase-2	0.47	↓	0.0313
P48500	Triosephosphate isomerase	0.46	↓	0.0185
P49432	Pyruvate dehydrogenase E1 component subunit beta, mitochondrial	0.46	↓	0.0320
Q9JMI1	Acetoacetyl-CoA synthetase	0.46	↓	0.0234
P27867	Sorbitol dehydrogenase	0.46	↓	0.0189
P61107	Ras-related protein Rab-14	0.46	↓	0.0011
P31016	Disks large homolog 4	0.46	↓	0.0103
Q03344	A TPase inhibitor, mitochondrial	0.46	↓	0.0479
P04642	L-lactate dehydrogenase A chain	0.45	↓	0.0060

Continued Table

UniProt IDs	Protein names	Fold change		*P*-value
Q5U316	Ras-related protein Rab-35	0.45	↓	0.0000
P62839	Ubiquitin-conjugating enzyme E2 D2	0.45	↓	0.0318
A8IHN8	Uncharacterized protein C11orf96 homolog	0.45	↓	0.0001
O70351	3-hydroxyacyl-CoA dehydrogenase type-2	0.45	↓	0.0359
P62632	Elongation factor 1-alpha 2	0.45	↓	0.0006
Q9WTV5	26S proteasome non-ATPase regulatory subunit 9	0.45	↓	0.0105
P11232	Thioredoxin	0.45	↓	0.0026
P13084	Nucleophosmin	0.45	↓	0.0018
P62824	Ras-related protein Rab-3C	0.45	↓	0.0293
P52481	Adenylyl cyclase-associated protein 2	0.45	↓	0.0043
P54290	Voltage-dependent calcium channel subunit alpha-2/delta-1	0.45	↓	0.0266
Q5M819	Phosphoserine phosphatase	0.45	↓	0.0042
Q6XVN8	Microtubule-associated proteins 1A/1B light chain 3A	0.45	↓	0.0024
P24329	Thiosulfate sulfurtransferase	0.44	↓	0.0018
Q9Z1N4	3'(2'), 5'-bisphosphate nucleotidase 1(Bpnt1)	0.44	↓	0.0083
P17702	60S ribosomal protein L28	0.44	↓	0.0404
O88767	Protein/nucleic acid deglycase DJ-1	0.44	↓	0.0002
P85972	Vinculin	0.44	↓	0.0145
P32551	Cytochrome b-c1 complex subunit 2, mitochondrial	0.44	↓	0.0158
P45592	Cofilin-1	0.44	↓	0.0000
Q569B7	RWD domain-containing protein 4	0.44	↓	0.0062
P02401	60S acidic ribosomal protein P2	0.44	↓	0.0322
Q7TT49	Serine/threonine-protein kinase MRCK beta	0.43	↓	0.0265
Q5PPJ9	Endophilin-B2	0.43	↓	0.0066

Continued Table

UniProt IDs	Protein names	Fold change		*P*-value
P40307	Proteasome subunit beta type-2	0.43	↓	0.0002
Q9QYU4	Ketimine reductase mu-crystallin	0.43	↓	0.0334
O70441	Synapsin-3	0.43	↓	0.0381
P36876	Serine/threonine-protein phosphatase 2A 55 kDa regulatory subunit B alpha isoform	0.43	↓	0.0038
Q9JLJ3	4-trimethylaminobutyraldehyde dehydrogenase	0.43	↓	0.0028
O35346	Focal adhesion kinase 1	0.42	↓	0.0176
P47875	Cysteine and glycine-rich protein 1	0.42	↓	0.0001
P10860	Glutamate dehydrogenase 1, mitochondrial	0.42	↓	0.0001
P70550	Ras-related protein Rab-8B	0.42	↓	0.0038
P40241	CD9 antigen	0.42	↓	0.0207
Q6AYK8	Eukaryotic translation initiation factor 3 subunit D	0.42	↓	0.0005
Q792I0	Protein lin-7 homolog C	0.42	↓	0.0003
P20651	Serine/threonine-protein phosphatase 2B catalytic subunit beta isoform	0.41	↓	0.0018
Q6P9U8	Eukaryotic translation initiation factor 3 subunit H	0.41	↓	0.0067
Q68FU3	Electron transfer flavoprotein subunit beta	0.41	↓	0.0025
P85845	Fascin	0.41	↓	0.0048
Q64559	Cytosolic acyl coenzyme A thioester hydrolase	0.41	↓	0.0069
P19132	Ferritin heavy chain	0.41	↓	0.0026
A1L108	Actin-related protein 2/3 complex subunit 5-like protein	0.41	↓	0.0045
E9PU28	Inosine-5'-monophosphate dehydrogenase 2	0.40	↓	0.0200
P38659	Protein disulfide-isomerase A4	0.40	↓	0.0376
P50554	4-aminobutyrate aminotransferase, mitochondrial	0.40	↓	0.0070
P18297	Sepiapterin reductase	0.40	↓	0.0001
Q5GFD9	Protein IMPACT	0.40	↓	0.0001

Continued Table

UniProt IDs	Protein names	Fold change		*P*-value
P23965	Enoyl-CoA delta isomerase 1, mitochondrial	0.36	↓	0.0087
P18421	Proteasome subunit beta type-1	0.36	↓	0.00231
P13676	Acylamino-acid-releasing enzyme	0.36	↓	0.0148
Q5U2Z3	Nucleosome assembly protein 1-like 4	0.36	↓	0.0226
Q9R0I8	Phosphatidylinositol 5-phosphate 4-kinase type-2 alpha	0.36	↓	0.0248
Q9Z339	Glutathione S-transferase omega-1	0.36	↓	0.0028
P23928	Alpha-crystallin B chain	0.35	↓	0.0064
Q9Z0G8	WAS/WASL-interacting protein family member	0.35	↓	0.0047
Q5U2Q7	Eukaryotic peptide chain release factor subunit 1	0.35	↓	0.0360
Q6PDV7	60S ribosomal protein L10	0.35	↓	0.0007
Q9EQV6	Tripeptidyl-peptidase 1	0.35	↓	0.0193
Q66HA5	Coiled-coil and C2 domain-containing protein 1A	0.35	↓	0.0132
Q6P7Q4	Lactoylglutathione lyase	0.35	↓	0.0089
Q6AY09	Heterogeneous nuclear ribonucleoprotein H2	0.35	↓	0.0029
P63102	14-3-3 protein zeta/delta	0.35	↓	0.0001
P34067	Proteasome subunit beta type-4	0.35	↓	0.0008
P21708	Mitogen-activated protein kinase 3	0.34	↓	0.0127
Q5HZV9	Protein phosphatase 1 regulatory subunit 7	0.34	↓	0.0003
Q5MJ12	F-box/LRR-repeat protein 16	0.34	↓	0.0035
P23978	Sodium- and chloride-dependent GABA transporter 1	0.34	↓	0.0390
P10760	Adenosylhomocysteinase	0.34	↓	0.0009
Q6P7A9	Lysosomal alpha-glucosidase	0.34	↓	0.0015
Q9WVC0	Septin-7	0.34	↓	0.0054

Continued Table

UniProt IDs	Protein names	Fold change		*P*-value
A9CMA6	Transmembrane protein 163	0.34	↓	0.0160
P20280	60S ribosomal protein L21	0.34	↓	0.0241
Q9Z2F5	C-terminal-binding protein 1	0.34	↓	0.0002
Q4V7C7	Actin-related protein 3	0.34	↓	0.0009
Q64057	Alpha-aminoadipic semialdehyde dehydrogenase	0.34	↓	0.0001
P31399	ATP synthase subunit d, mitochondrial	0.34	↓	0.0284
P62912	60S ribosomal protein L32	0.33	↓	0.0304
B2RZ37	Receptor expression-enhancing protein 5	0.33	↓	0.0020
P68101	Eukaryotic translation initiation factor 2 subunit 1	0.33	↓	0.0030
P51650	Succinate-semialdehyde dehydrogenase, mitochondrial	0.33	↓	0.0003
P55063	Heat shock 70 kDa protein 1-like	0.33	↓	0.0260
Q3T1J1	Eukaryotic translation initiation factor 5A-1	0.33	↓	0.0004
P18445	60S ribosomal protein L27a	0.33	↓	0.0021
O35824	DnaJ homolog subfamily A member 2	0.33	↓	0.0183
Q6J2U6	E3 ubiquitin-protein ligase RNF114	0.33	↓	0.0355
P49242	40S ribosomal protein S3a	0.33	↓	0.0017
P62161	Calmodulin-2/Calmodulin-3/Calmodulin-1	0.32	↓	0.0023
P04762	Catalase	0.32	↓	0.0023
P63029	Translationally-controlled tumor protein	0.32	↓	0.0283
Q6JE36	Protein NDRG1	0.32	↓	0.0094
P35427	60S ribosomal protein L13a	0.32	↓	0.0040
Q62688	Inactive phospholipase C-like protein 1	0.32	↓	0.0047
Q8R4A1	ERO1-like protein alpha	0.32	↓	0.0087

Continued Table

UniProt IDs	Protein names	Fold change		*P*-value
P04644	40S ribosomal protein S17	0.32	↓	0.0249
P84092	AP-2 complex subunit mu	0.31	↓	0.0002
Q9R085	Ubiquitin carboxyl-terminal hydrolase 15	0.31	↓	0.0004
P60522	Gamma-aminobutyric acid receptor-associated protein-like 2	0.31	↓	0.0010
Q5PPN5	Tubulin polymerization-promoting protein family member 3	0.31	↓	0.0016
P62828	GTP-binding nuclear protein Ran	0.31	↓	0.0000
P14668	Annexin A5	0.31	↓	0.0001
Q5RKI1	Eukaryotic initiation factor 4A-II	0.31	↓	0.0025
Q9Z142	Transmembrane protein 33	0.31	↓	0.0087
Q6GMN2	Brain-specific angiogenesis inhibitor 1-associated protein 2	0.31	↓	0.0048
P08082	Clathrin light chain B	0.30	↓	0.0009
Q9JJ54	Heterogeneous nuclear ribonucleoprotein D0	0.30	↓	0.0002
P62909	40S ribosomal protein S3	0.30	↓	0.0103
Q9WTT6	Guanine deaminase	0.30	↓	0.0003
Q9JKE3	Secretory carrier-associated membrane protein 5	0.30	↓	0.0186
P07943	Aldose reductase	0.30	↓	0.0023
Q9JIX3	Bis	0.30	↓	0.0105
Q9Z269	V esicle-associated membrane protein-associated protein B	0.30	↓	0.0112
Q9JHW0	Proteasome subunit beta type-7	0.29	↓	0.0270
O09032	ELAV-like protein 4	0.29	↓	0.0053
Q5U2R7	LRP chaperone MESD	0.29	↓	0.0000
Q9WU34	Neuronal-specific septin-3	0.29	↓	0.0002

Continued Table

UniProt IDs	Protein names	Fold change		*P*-value
P22734	Catechol O-methyltransferase	0.29	↓	0.0145
P0C2X9	Delta-1-pyrroline-5-carboxylate dehydrogenase, mitochondria	0.29	↓	0.0088
P19139	Casein kinase II subunit alpha	0.29	↓	0.0073
P53678	AP-3 complex subunit mu-2	0.29	↓	0.0025
P05712	Ras-related protein Rab-2A	0.29	↓	0.0015
P17764	Acetyl-CoA acetyltransferase, mitochondrial	0.28	↓	0.0009
Q5EGY4	Synaptobrevin homolog YKT6	0.28	↓	0.0001
Q6AYN4	Phytanoyl-CoA hydroxylase-interacting protein-like	0.28	↓	0.0001
Q7M0E3	Destrin	0.28	↓	0.0010
P15791	Calcium/calmodulin-dependent protein kinase type II subunit delta	0.28	↓	0.0098
Q9Z0W7	Chloride intracellular channel protein 4	0.28	↓	0.0161
Q4KLZ6	Triokinase/FMN cyclase	0.28	↓	0.0035
P14173	Aromatic-L-amino-acid decarboxylase	0.27	↓	0.0000
Q5FVJ0	Protein RUFY3	0.27	↓	0.0001
O35331	Pyridoxal kinase	0.27	↓	0.0310
O55166	V acuolar protein sorting-associated protein 52 homolog	0.27	↓	0.0042
P19234	NADH dehydrogenase [ubiquinone] flavoprotein 2, mitochondrial	0.27	↓	0.0047
Q498R7	UPF0587 protein C1orf123 homolog	0.27	↓	0.0016
P62859	40S ribosomal protein S28	0.27	↓	0.0005
P46844	Biliverdin reductase A	0.27	↓	0.0074
P31232	Transgelin	0.27	↓	0.0001
O35828	Coronin-7	0.27	↓	0.0047

Continued Table

UniProt IDs	Protein names	Fold change		*P*-value
Q63569	26S proteasome regulatory subunit 6A	0.27	↓	0.0000
Q78P75	Dynein light chain 2, cytoplasmic	0.27	↓	0.0080
Q9R063	Peroxiredoxin-5, mitochondrial	0.27	↓	0.0000
Q6AYU3	DnaJ homolog subfamily B member 6	0.26	↓	0.0068
P04182	Ornithine aminotransferase, mitochondrial	0.26	↓	0.0025
Q4G017	Nischarin	0.26	↓	0.0000
P11506	Plasma membrane calcium-transporting A TPase 2	0.26	↓	0.0012
P04903	Glutathione S-transferase alpha-2	0.26	↓	0.0003
P35281	Ras-related protein Rab-10	0.26	↓	0.0008
P84817	Mitochondrial fission 1 protein	0.26	↓	0.0264
P02091	Hemoglobin subunit beta-1	0.26	↓	0.0017
P70645	Bleomycin hydrolase	0.25	↓	0.0000
P29411	GTP:AMP phosphotransferase AK3, mitochondrial	0.25	↓	0.0011
P48037	Annexin A6	0.25	↓	0.0006
P15651	Short-chain specific acyl-CoA dehydrogenase, mitochondrial	0.25	↓	0.0012
F1LNI5	Protein phosphatase 1G	0.25	↓	0.0427
Q5HZY2	GTP-binding protein SAR1b	0.25	↓	0.0069
Q9QUR2	Dynactin subunit 4	0.25	↓	0.0248
P48199	C-reactive protein	0.25	↓	0.0020
Q9JJM9	Septin-5	0.24	↓	0.0014
P04905	Glutathione S-transferase Mu 1	0.24	↓	0.0001
P09527	Ras-related protein Rab-7a	0.24	↓	0.0001
P59215	Guanine nucleotide-binding protein G	0.24	↓	0.0012

Continued Table

UniProt IDs	Protein names	Fold change		*P*-value
P04646	60S ribosomal protein L35a	0.23	↓	0.0002
P18422	Proteasome subunit alpha type-3	0.23	↓	0.0012
B2RYW9	Fumarylacetoacetate hydrolase domain-containing protein 2	0.23	↓	0.0000
Q63622	Disks large homolog 2	0.23	↓	0.0017
Q6MG48	Protein PRRC2A	0.23	↓	0.0326
Q9QX69	LanC-like protein 1	0.23	↓	0.0015
P60901	Proteasome subunit alpha type-6	0.23	↓	0.0014
P01830	Thy-1 membrane glycoprotein	0.23	↓	0.0027
O88637	Ethanolamine-phosphate cytidylyltransferase	0.23	↓	0.0050
P12749	60S ribosomal protein L26	0.22	↓	0.0002
Q9WUS0	Adenylate kinase 4, mitochondrial	0.22	↓	0.0000
Q9ESI7	Neuronal migration protein doublecortin	0.22	↓	0.0025
P02770	Serum albumin	0.22	↓	0.0000
P19643	Amine oxidase [flavin-containing] B	0.22	↓	0.0112
P14604	Enoyl-CoA hydratase, mitochondrial	0.21	↓	0.0073
P02767	Transthyretin	0.21	↓	0.0001
P14659	Heat shock-related 70 kDa protein 2	0.21	↓	0.0020
Q9EPB1	Dipeptidyl peptidase 2	0.21	↓	0.0000
P00507	Aspartate aminotransferase, mitochondrial	0.21	↓	0.0007
P48004	Proteasome subunit alpha type-7	0.21	↓	0.0047
P07895	Superoxide dismutase [Mn], mitochondrial	0.21	↓	0.0018
P68136	Actin, alpha skeletal muscle	0.21	↓	0.0007

Continued Table

UniProt IDs	Protein names	Fold change		*P*-value
Q4KM65	Cleavage and polyadenylation specificity factor subunit 5	0.20	↓	0.0139
Q6AYD3	Proliferation-associated protein 2G4	0.20	↓	0.0013
P28818	Ras-specific guanine nucleotide-releasing factor 1	0.20	↓	0.0251
P04937	Fibronectin	0.20	↓	0.0023
Q01986	Dual specificity mitogen-activated protein kinase kinase 1	0.20	↓	0.0032
P08009	Glutathione S-transferase Yb-3	0.20	↓	0.0005
P35213	14-3-3 protein beta/alpha	0.20	↓	0.0057
B0BN93	26S proteasome non-A TPase regulatory subunit 13	0.20	↓	0.0021
P62076	Mitochondrial import inner membrane translocase subunit Tim13	0.20	↓	0.0041
P11348	Dihydropteridine reductase	0.20	↓	0.0001
P61203	COP9 signalosome complex subunit 2	0.20	↓	0.0001
Q9Z214	Homer protein homolog 1	0.20	↓	0.0080
Q64361	Latexin	0.20	↓	0.0149
Q6AYS7	Aminoacylase-1A	0.19	↓	0.0004
Q9Z1B2	Glutathione S-transferase Mu 5	0.19	↓	0.0008
P63045	Vesicle-associated membrane protein 2	0.19	↓	0.0199
Q05962	ADP/ATP translocase 1	0.19	↓	0.0009
P69897	Tubulin beta-5 chain	0.19	↓	0.0001
Q9WTT7	Basic leucine zipper and W2 domain-containing protein 2	0.19	↓	0.0154
P60892	Ribose-phosphate pyrophosphokinase 1	0.19	↓	0.0045
Q5BJR4	Protein prune homolog 2	0.19	↓	0.0000

Continued Table

UniProt IDs	Protein names	Fold change		*P*-value
Q32Q06	AP-1 complex subunit mu-1	0.19	↓	0.0000
Q5BK81	Prostaglandin reductase 2	0.19	↓	0.0002
Q9WVB1	Ras-related protein Rab-6A	0.19	↓	0.0488
Q4FZT2	Protein phosphatase methylesterase 1	0.19	↓	0.0000
P28075	Proteasome subunit beta type-5	0.19	↓	0.0014
P25093	Fumarylacetoacetase	0.19	↓	0.0028
P05696	Protein kinase C alpha type	0.19	↓	0.0001
Q63525	Nuclear migration protein nudC	0.18	↓	0.0030
Q63416	Inter-alpha-trypsin inhibitor heavy chain H3	0.18	↓	0.0003
P30904	Macrophage migration inhibitory factor	0.18	↓	0.0022
Q91Y81	Septin-2	0.18	↓	0.0023
Q6DGG1	Protein ABHD14B	0.18	↓	0.0022
Q641X8	Eukaryotic translation initiation factor 3 subunit E	0.18	↓	0.0002
P15178	Aspartate--tRNA ligase, cytoplasmic	0.18	↓	0.0009
P12346	Serotransferrin	0.18	↓	0.0042
O88989	Malate dehydrogenase, cytoplasmic	0.18	↓	0.0037
P68255	14-3-3 protein theta	0.18	↓	0.0011
P49187	Mitogen-activated protein kinase 10	0.18	↓	0.0001
P17425	Hydroxymethylglutaryl-CoA synthase, cytoplasmic	0.18	↓	0.0110
P84850	D-2-hydroxyglutarate dehydrogenase, mitochondrial	0.17	↓	0.0060
P29315	Ribonuclease inhibitor	0.17	↓	0.0007
Q5HZE4	Methylthioribose-1-phosphate isomerase	0.17	↓	0.0263
P85973	Purine nucleoside phosphorylase	0.17	↓	0.0027

Continued Table

UniProt IDs	Protein names	Fold change		*P*-value
P63012	Ras-related protein Rab-3A	0.17	↓	0.0138
Q5PQN7	Protein LZIC	0.17	↓	0.0007
P21670	Proteasome subunit alpha type-4	0.17	↓	0.0010
P68370	Tubulin alpha-1A chain	0.17	↓	0.0001
P10888	Cytochrome c oxidase subunit 4 isoform 1, mitochondrial	0.17	↓	0.0015
P01946	Hemoglobin subunit alpha-1/2	0.16	↓	0.0136
Q63942	GTP-binding protein Rab-3D	0.16	↓	0.0001
O35764	Neuronal pentraxin receptor	0.16	↓	0.0001
Q6P4Z6	Leucine carboxyl methyltransferase 1	0.16	↓	0.0144
B0BNM9	Glycolipid transfer protein	0.16	↓	0.0015
P63245	Receptor of activated protein C kinase 1	0.15	↓	0.0000
P27605	Hypoxanthine-guanine phosphoribosyltransferase	0.15	↓	0.0016
P47728	Calretinin	0.15	↓	0.0010
P55051	Fatty acid-binding protein, brain	0.15	↓	0.0002
P29266	3-hydroxyisobutyrate dehydrogenase, mitochondrial	0.14	↓	0.0200
P70566	Tropomodulin-2	0.14	↓	0.0005
P16036	Phosphate carrier protein, mitochondrial	0.14	↓	0.0092
P19945	60S acidic ribosomal protein P0	0.14	↓	0.0005
P84083	ADP-ribosylation factor 5	0.14	↓	0.0215
O35509	Ras-related protein Rab-11B	0.14	↓	0.0013
P20761	Ig gamma-2B chain C region	0.14	↓	0.0050
Q9Z2L0	Voltage-dependent anion-selective channel protein 1	0.14	↓	0.0000
P11517	Hemoglobin subunit beta-2	0.14	↓	0.0005

Continued Table

UniProt IDs	Protein names	Fold change		*P*-value
P08523	Olfactory marker protein	0.13	↓	0.0047
Q9Z0V6	Thioredoxin-dependent peroxide reductase, mitochondrial	0.13	↓	0.0342
Q3B8Q2	Eukaryotic initiation factor 4A-III	0.13	↓	0.0004
Q63941	Ras-related protein Rab-3B	0.13	↓	0.0009
P23562	Band 3 anion transport protein	0.13	↓	0.0004
P62260	14-3-3 protein epsilon	0.13	↓	0.0024
P61354	60S ribosomal protein L27	0.13	↓	0.0001
P62762	Visinin-like protein 1	0.12	↓	0.0040
P32851	Syntaxin-1A	0.12	↓	0.0117
P70470	Acyl-protein thioesterase 1	0.11	↓	0.0000
Q5I0D1	Glyoxalase domain-containing protein 4	0.11	↓	0.0081
P17078	60S ribosomal protein L35	0.11	↓	0.0016
P62853	40S ribosomal protein S25	0.11	↓	0.0020
P56536	Kinesin heavy chain isoform 5C	0.11	↓	0.0143
P24268	Cathepsin D	0.11	↓	0.0049
Q62818	Translation initiation factor eIF-2B subunit beta	0.10	↓	0.0127
P19804	Nucleoside diphosphate kinase B	0.10	↓	0.0209
P38062	Methionine aminopeptidase 2	0.10	↓	0.0000
P62278	40S ribosomal protein S13	0.10	↓	0.0084

Continued Table

UniProt IDs	Protein names	Fold change		*P*-value
Q68FS2	COP9 signalosome complex subunit 4	0.10	↓	0.0006
Q99PD4	Actin-related protein 2/3 complex subunit 1A	0.10	↓	0.0020
Q5RJL0	Ermin	0.10	↓	0.0001
Q7TP52	Carboxymethylenebutenolidase homolog	0.09	↓	0.0259
Q5XIC0	Enoyl-CoA delta isomerase 2, mitochondrial	0.09	↓	0.0133
Q62876	Synaptogyrin-1	0.09	↓	0.0130
Q9WU49	Calcium-regulated heat stable protein 1	0.09	↓	0.0004
Q63396	Activated RNA polymerase II transcriptional coactivator p15	0.08	↓	0.0015
P18437	Non-histone chromosomal protein HMG-17	0.08	↓	0.0044
P05371	Clusterin	0.08	↓	0.0009
P0C0A1	V acuolar protein-sorting-associated protein 25	0.07	↓	0.0007
Q5PQN1	Probable E3 ubiquitin-protein ligase HERC4	0.07	↓	0.0048
P54313	Guanine nucleotide-binding protein G	0.07	↓	0.0052
P63004	Platelet-activating factor acetylhydrolase IB subunit alpha	0.07	↓	0.0152
P62630	Elongation factor 1-alpha 1	0.06	↓	0.0003
Q09073	ADP/ATP translocase 2	0.06	↓	0.0028
P05714	Ras-related protein Rab-4A	0.06	↓	0.0002
Q6NYB7	Ras-related protein Rab-1A	0.06	↓	0.0026
P35280	Ras-related protein Rab-8A	0.06	↓	0.0002

Continued Table

UniProt IDs	Protein names	Fold change		*P*-value
Q8K4F7	m7GpppX diphosphatase	0.06	↓	0.0303
Q568Z6	IST1 homolog	0.06	↓	0.0001
Q4AE70	Histone-arginine methyltransferase CARM1	0.05	↓	0.0002
P63074	Eukaryotic translation initiation factor 4E	0.05	↓	0.0038
B1H267	Sorting nexin-5	0.05	↓	0.0028
P02651	Apolipoprotein A-IV	0.05	↓	0.0038
Q6AXT5	Ras-related protein Rab-21	0.05	↓	0.0064
Q920P0	L-xylulose reductase	0.05	↓	0.0000
P48508	Glutamate--cysteine ligase regulatory subunit	0.04	↓	0.0276
Q6AY56	Tubulin alpha-8 chain	0.04	↓	0.0004
Q812E9	Neuronal membrane glycoprotein M6-a	0.04	↓	0.0010
P01015	Angiotensinogen	0.04	↓	0.0001
P83953	Importin subunit alpha-5	0.04	↓	0.0151
P19511	ATP synthase F	0.03	↓	0.0054
P22057	Prostaglandin-H2 D-isomerase	0.03	↓	0.0000
P05544	Serine protease inhibitor A3L	0.01	↓	0.0003
P14046	Alpha-1-inhibitor 3	0.03	↓	0.0049
Q63041	Alpha-1-macroglobulin	0.02	↓	0.0219
O08618	Phosphoribosyl pyrophosphate synthase-associated protein 2	0.02	↓	0.0002
Q66HG4	Aldose 1-epimerase	0.02	↓	0.0029
P85970	Actin-related protein 2/3 complex subunit 2	0.01	↓	0.0008
P13635	Ceruloplasmin	0.01	↓	0.0001
P01026	Complement C3	0.01	↓	0.0002
P17475	Alpha-1-antiproteinase	0.01	↓	0.0053
